# Assessment of Indonesian-Language Orthodontics-Related YouTube Video as a Source of Information

**DOI:** 10.1055/s-0042-1744375

**Published:** 2022-07-14

**Authors:** Ninuk Hariyani, Aurellia Rahmawati, Yuanita Lely Rachmawati, Anton Rahardjo, Diah Ayu Maharani

**Affiliations:** 1 Department of Dental Public Health, Faculty of Dental Medicine, Universitas Airlangga, Surabaya, Indonesia; 2 Department of Preventive and Public Health Dentistry, Faculty of Dentistry, Universitas Indonesia, Jakarta, Indonesia; 3 Department of Community and Preventive Dentistry, Faculty of Dentistry, Universitas Brawijaya, Malang, Indonesia

**Keywords:** Indonesia, orthodontics, videos, YouTube, internet

## Abstract

**Objectives** Health-seeking behavior through social media including orthodontics treatment has become popular among community in Indonesia. However, the characteristics of uploaded video in term of quality, reliability, and usefulness are unknown. This study aims to analyze the characteristics of Indonesian-language orthodontic-related YouTube videos uploaded.

**Materials and Methods** This study adopted cross-sectional design and analyzed 300 videos as the sample. A final 100 related videos were included for analysis of the quality, usefulness, and reliability of the video uploaded as well as viewers' interaction in term of popularity and visibility. Mann–Whitney's test was used for the statistical analysis.

**Results** The majority of the videos were uploaded by individual users (60%) with moderate quality, usefulness, and reliability. Statistical analysis showed that orthodontics-related YouTube videos uploaded by individuals have lower popularity and reliability compared with health professional ( *p* < 0.05).

**Conclusion** Most of the Indonesian-language orthodontic-related YouTube videos have moderate quality, usefulness, and reliability. There is a need from health professional to contribute more related video as main source of health information for the general to make healthy health-seeking behavior.

## Introduction

Internet is useful for learning tools and also spreading health-related information and disease prevention campaign. 1 2 Learning, communicating, and disseminating reliable and useful health information using the internet became more important in the pandemic era where dentists and patients are concerned for the pandemic's effects. 3 4 Information on the internet can be used as a complement to complete information provided by health professionals, family, friends, and other traditional media. 5 Easy access to health-related information is expected to increase patients' active role in decision-making along with health professionals. 6 Health-seeking information on social media is not only for knowledge purposes but also to gain social and emotional support from other social media users. 7 Even though social media could be used for health communication, the information shared sometimes lacks reliability. 8 Thus, the quality and reliability of the information should be monitored to ensure that users received correct information they need. 8

Social media usage, such as video, provides chances to gain and assess contents that are relevant and could be trusted by the users. 9 Nowadays, free sites that are usually used for spreading information through video are Google, Facebook, and YouTube. 10 11 The use of social media in dentistry is increasing, including on YouTube. 12 13 Validity and accuracy are some points that we have to be cautious about when we use information on YouTube as a source of health information. Research has shown that the increasing usage of YouTube affects patients' decision-making in many aspects of dental health, including in dental esthetics. 10 14 15

Nowadays, esthetics is a popular topic in many fields, both in dentistry and in modern society. 16 One of the important elements in esthetics assessment is the dental and oral condition, which is considered to provide social benefits. Dental esthetics could affect others' perceptions and yourself. 17 18 Dental esthetics has a bigger impact on individual's psychological status than oral, physical, or functional status domains from oral health-related quality of life. 17 Perfectly esthetic teeth are considered as a self-esteem booster in social life. 17 19 There is a presumption on an interpersonal relationship where untreated teeth are a form of indifference to self. 19 Research showed that patient urges to seek an orthodontic treatment as the patient wants to increase his/her smile esthetics that could give a bigger impact on his/her social life. 20 Prevalence of malocclusion in Indonesia is very high, around 80% of the population. 21 Patients' desire to enhance the esthetics of their smile prompted them to undertake orthodontic treatment. 20 Orthodontic treatment is a treatment that focuses on correcting the position of the teeth and correcting malocclusion. Many patients want to undergo orthodontic treatment to improve their appearance and to be accepted in society rather than to improve their teeth and oral function or health. 22

Previous studies have been conducted to learn the use of video in providing health-related information. 10 23 A previous study assessed the quality of videos related to dental implant by using usefulness score and concluded that YouTube videos related to dental implant were a limited source for the patients. 10 Study on videos about burning mouth syndrome showed that information on YouTube is less reliable and not based on science. 23 Furthermore, research assessing orthodontic-related videos showed that YouTube may provide an opportunity for orthodontic professionals to disseminate health information. 14 15

However, the majority of these studies assessed videos in English language, while the study on Indonesian-language videos is scarce. As Indonesia is the fourth most populated country in the world after China, India, and the United States, with Indonesian being used by more than 94% of the population, 24 there is a need to report the characteristics of Indonesian-language videos. In Indonesia, there is still a lack of information about the characteristics of Indonesian-language videos on YouTube as a media of information about orthodontics. Thus, this study aims to assess the characteristics of Indonesian-language videos on YouTube as a media of information related to orthodontics.

## Materials and Methods

### YouTube Search Strategy

**Fig. 1:**
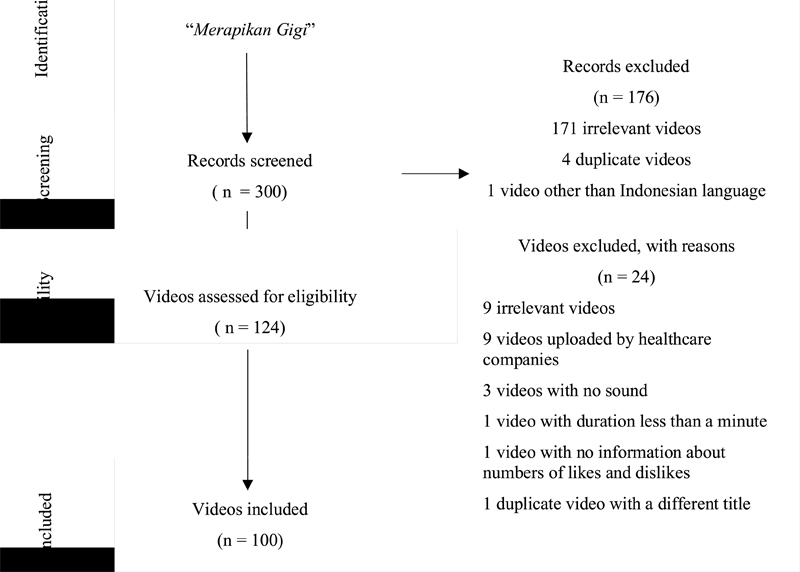
Video selection for analysis using Preferred Reporting Items for Systematic Reviews and Meta-Analyses flow diagram.

Orthodontics-related video in Indonesian language uploaded on YouTube in the last 12 months were searched and screened ( www.youtube.com ) on September 21, 2020, by using “sort by relevance” filter. The search term used was “merapikan gigi,” a general term for orthodontic treatment in Indonesian language. We took the first 300 videos from the search results. Video selection for analysis followed the flow diagram ( Fig. 1 ). Videos' links were saved for further analyses. 25

### Video Selection Criteria

Videos were sorted according to inclusion and exclusion criteria as shown in Fig. 1 . The inclusion criteria were Indonesian-language orthodontic-related video. Initial screening of videos was performed to exclude videos in languages other than Indonesian; videos with no sound or headings; duplicate videos; irrelevant videos such as celebrity news; videos about other types of dental treatments; videos that were uploaded by health companies or advertisements; videos with duration less than a minute; and videos without information about the numbers of likes and dislikes.

### Analysis of Videos

**Fig. 2:**
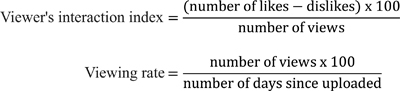
The interaction index and the viewing rate formulas.

Two researchers (A.R. and D.A.M.) were independently assessed videos. Information extracted included the video's uploader, date of upload, the number of views, likes, dislikes, and duration of each video. The uploaders were categorized as individual users and health care professionals. Viewers' interactions with videos were evaluated based on the viewer's interaction index (popularity) and the viewing rate (visibility) formulas, as presented in Fig. 2 . 25 26

The quality, uniqueness, and reliability of the video were assessed using the Global Quality Scale, 27 usefulness score, 10 and DISCERN questionnaire, 28 respectively. For objective assessment, every researcher scored videos independently. In case of disagreement regarding the score, a third reviewer's opinion was sought for further discussion and a decision was made by consensus.

The quality of the videos was classified according to criteria proposed by Sorensen et al 27 as follows: 1 = very poor quality, poor flow, lack of information, and nothing useful to patients; 2 = generally poor quality, low level of flow, some information are listed but few important topics are addressed, very limited use for patients; 3 = moderate quality, flow below ideal, some important information are adequately discussed, but other pieces of information are poorly discussed, somewhat useful to patients; 4 = good quality, generally good flow, most of the relevant information are listed, but some topics are not addressed, useful to patients; and 5 = excellent quality, excellent flow, very useful to patients. 26

The evaluation of the video's usefulness score was based on the content presence in eight nonmutually exclusive domains of information on orthodontics. Those domains include definition, indications, contraindications, advantages, procedures involved, complications, prognosis and survival, and cost. 10 One point was given for each contents and the total maximum score was eight. A score of 0 to 2 indicated poor video content that composed misleading information and whose information about eight domains evaluated was not all useful; a score of 3 to 5 indicated moderate video content that gave a positive message related to orthodontic treatment but poorly discussed some domains; a score of 6 to 8 showed excellent video content that gave detailed, valid, and correct information for patients. 10

In analyzing the reliability of the videos, a questionnaire by Singh et al was used. 28 For each aspect addressed, videos received 1 point, with possible scores ranging from 0 to 5 points. The criteria used in this analysis were as follows: (1) Are objectives clear and achieved? (2) Are the sources of information used reliable? (3) Is the information presented balanced and unbiased? (4) Are additional sources of information listed for patient reference? (5) Are areas of uncertainty mentioned? 28

### Statistical Analysis

The research data of the video characteristics were collected in Microsoft Excel, then processed and analyzed using IBM SPSS 22.0. The inter- and intraobserver reliability tests were conducted with the intraclass correlation coefficient. Descriptive analysis using mean, median, and frequency was conducted to give general information related to the characteristics of video uploaded. The normality test and the Mann–Whitney's test were performed to determine the differences between video categories. The statistical significance was evaluated at *p* < 0.05.

## Results

The first 300 videos by relevance were added to a YouTube playlist. Videos were screened by its title and there were 176 videos excluded because of irrelevant titles and duplication. There were 124 videos assessed and 24 videos were further excluded. The exclusion was because the videos were irrelevant (the title and content do not match), uploaded by health companies, without sound, and less than 1 minute in duration, had no information on likes and dislikes, and had duplicate contents with different titles. Therefore, the remaining 100 videos were included ( Fig. 1 ).

The reliability tests of 20 videos by two observers indicated excellent inter- and intra-observer agreement (>0.80). Table 1 shows the characteristics of the analyzed videos. The majority of the sample were videos uploaded by individual users ( *n* = 60, 60%) with the average duration of 493 seconds (ranging from 76 to 2,920 seconds). The least viewed video had 71 views and the most viewed video had 847,395 views. The most popular video had 21,891 likes and 325 dislikes. The videos analyzed averagely had moderate quality and moderate usefulness.

**Table 1:** Characteristics of Indonesian-language YouTube orthodontics videos

Variable	
Uploader, *n* (%)	
Individual users	60 (60%)
Health care professionals	40 (40%)
Number of days of upload (d)	
Mean (SD)	172 (103)
Median (minimum–maximum)	166 (5–365)
Duration (s)	
Mean (SD)	493 (361)
Median (minimum–maximum)	415 (76–2,920)
Views	
Mean (SD)	30,859 (103,033)
Median (minimum–maximum)	5,115 (71–847,395)
Likes	
Mean (SD)	542 (2,377)
Median (minimum–maximum)	83 (1–21,891)
Dislikes	
Mean (SD)	16 (40)
Median (minimum–maximum)	3 (0–325)
Popularity	
Mean (SD)	2.37 (2.20)
Median (minimum–maximum)	1.67 (0.22–15.90)
Visibility	
Mean (SD)	14,062 (35,787)
Median (minimum–maximum)	3,413 (44–267,317)
Quality	
Mean (SD)	2.54 (1.15)
Median (minimum–maximum)	3 (1–5)
Usefulness	
Mean (SD)	3.18 (1.67)
Median (minimum–maximum)	3 (0–7)
Reliability	
Mean (SD)	2.57 (0.82)
Median (minimum–maximum)	3 (0–4)

Abbreviation: SD, standard deviation.

**Fig. 3:**
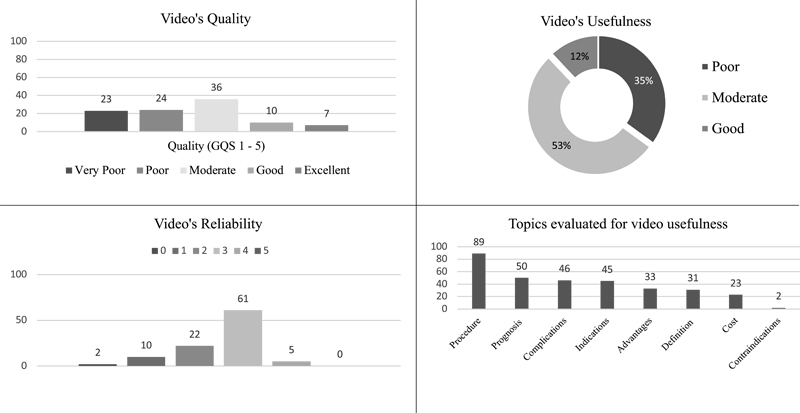
Characteristics of quality, usefulness, and reliability of YouTube orthodontics video.

Fig. 3 shows characteristics of quality, usefulness, and reliability of Indonesian-language orthodontic-related YouTube video. The majority of the videos have moderate quality, usefulness, and reliability. It shows that most of the orthodontic-related videos have insufficient information. A total of eight topic domains were assessed on the video. The most discussed topic was orthodontic procedures (89%). Meanwhile, the contraindication of the treatment was the least discussed topic (2%).

The characteristics of Indonesian-language orthodontic-related YouTube video based on its uploader and duration are presented in Table 2 . Videos uploaded by individual users and health professionals showed a significant difference of performance in popularity and reliability, in which video uploaded by health care professionals showed higher popularity and reliability ( *p* = 0.010 and *p* = 0.001, respectively). On the other hand, a significant higher difference was found for likes ( *p* = 0.027), popularity ( *p* = 0.001), quality ( *p* = 0.001), and usefulness ( *p* = 0.001) in video more than 6 minutes than less than 6 minutes length.

**Table 2:** Characteristics of Indonesian-language YouTube orthodontics video based on its uploader and duration

	Video's uploader		*p* -Value	Video's duration		*p* -Value
	Individual users ( *n* = 60)	Health care professionals ( *n* = 40)		Up to 6 min ( *n* = 16)	More than 6 min ( *n* = 84)	
	Median (minimum–maximum)	Median (minimum–maximum)		Median (minimum–maximum)	Median (minimum–maximum)	
Views	5,396 (134–847,395)	4,372 (71–329,905)	0.325	4,464 (71–417,005)	5,438 (296–847,395)	0.752
Visibility	4,204 (44–267,317)	2,769 (142–119,530)	0.325	2,845 (44–171,607)	4,422 (167–267,317)	0.311
Likes	72 (1–21,891)	119 (1–5,816)	0.673	61 (1–1,626)	120 (3–21,891)	0.027 a
Popularity	1.2 (0.2–7.0)	2.3 (0.2–15.9)	0.010 a	1.0 (0.2–8.4)	2.2 (0.2–15.9)	0.001 a
Quality	3.0 (1–5)	3.0 (1–5)	0.403	2.0 (1–5)	3.0 (1–5)	0.001 a
Usefulness	3.0 (0–7)	3.0 (0–7)	0.335	2.0 (0–6)	3.5 (0–7)	0.001 a
Reliability	2.5 (0–4)	3.0 (2–4)	0.001 a	3.0 (1–4)	3.0 (0–4)	0.264

Note: Mann–Whitney's test.

a Significant ( *p* -value <0.05).

Table 3 shows visibility and popularity of Indonesian-language orthodontic-related YouTube video based on video's quality, usefulness, and reliability. The popularity of videos with lower quality and higher quality differed significantly ( *p* = 0.001), in which videos with higher quality showed higher popularity. Videos with higher quality have a higher number of likes (108 likes vs. 68 likes; *p* = 0.405). However, videos with lower quality have a higher number of views (5,391 vs. 3,695 views; *p* = 0.401). In terms of video's usefulness, there were significant differences in visibility between videos with low and high usefulness (3,813 vs. 2,885 visibility; *p* = 0.008). Video with low usefulness have higher views and likes but lower popularity than high usefulness video (5,996 vs. 3,100 views [ *p* = 0.241]; 91 vs. 78 likes [ *p* = 0.655]; and 1.4 vs. 2.4 score popularity [ *p* = 0.390], respectively).Visibility and popularity of the videos were not different according to their reliability.

**Table 3:** Visibility and popularity differences based on video quality, video usefulness, and video reliability

	Video's quality		*p* -Value
	Qualities 1 and 2 ( *n* = 47)	Qualities 3–5 ( *n* = 53)	
	Median (minimum–maximum)	Median (minimum–maximum)	
Views	5,391 (134–417,005)	3,695 (71–847,395)	0.401
Visibility	3,842 (44–171,607)	3,073 (186–267,317)	0.636
Likes	68 (1–5,816)	108 (3–21,891)	0.405
Popularity	1.2 (0.2–6.5)	2.3 (0.5–15.9)	0.001 a
	Video's usefulness		*p* -Value
	Usefulness scores 0–3 ( *n* = 62)	Usefulness scores 4–8 ( *n* = 38)	
	Median (minimum–maximum)	Median (min–maximum)	
Views	5,996 (134–417,005)	3,100 (71–847,395)	0.241
Visibility	3,813 (44–171,607)	2,885 (186–267,317)	0.008 a
Likes	91 (1–5,816)	78 (6–21,891)	0.655
Popularity	1.4 (0.2–7.2)	2.4 (0.6–15.9)	0.390
	Video's reliability		*p* -Value
	Reliabilities 0–1 ( *n* = 12)	Reliabilities 2–5 ( *n* = 88)	
	Median (minimum–maximum)	Median (minimum–maximum)	
Views	10,056 (134–48,191)	4,517 (71–847,324)	0.178
Visibility	8,112 (44–22,228)	3,048 (142–267,174)	0.143
Likes	132 (1–710)	81 (1–21,891)	0.429
Popularity	1.2 (0.6–2.7)	1.9 (0.2–15.9)	0.075

Note: Mann–Whitney's test.

a Significant ( *p* -value <0.05).

Further analysis is presented in Appendices A and B . Appendix A shows the top 10 videos based on the total score of its quality, usefulness, and reliability. The video with the highest quality, usefulness, and reliability scores was the one uploaded by health care professionals. Appendix B shows the visibility and popularity of top 10 videos. Video with the highest popularity was also uploaded by health care professionals. However, the highest visibility in this study was in the videos uploaded by individual users.

## Discussion

YouTube, one of the social media platforms, has gained its popularity from people in seeking health-related information including orthodontics-related videos. 29 However, the accuracy, evidence based, and compliance are debatable. 30 Although plenty of orthodontics-related video has been posted for people to view in different languages, most of it uploaded by individual users 14 26 indicate a need of contribution from the health professionals. We found out that the number of Indonesian-language orthodontics video was also uploaded by individual users.

In this study, the majority of the orthodontic-related videos have moderate quality. Videos with a moderate quality show a below ideal flow, have some important information discussed, and are quite useful to the viewers. The majority of Indonesian-language orthodontic-related videos still have insufficient information but can be used as information that is quite useful to the audience. Our finding supported previous research about orthodontic-related videos in other language. 31 32 In making future videos, video uploaders can pay more attention to the video flow, information coverage, and benefits for the viewers. Thus, it is hoped that the resulting video will have good quality and be useful to viewers.

This study found that popularity and reliability were higher in videos uploaded by health professionals, supporting previous studies. 26 33 This indicates that viewers rate video uploaded by health care professionals as more trustworthy video. Furthermore, 53% of Indonesian-language orthodontic-related YouTube videos have a moderate usefulness score, which is better than the previous study. 10 These results indicate that the sufficiency of information in most Indonesian-language orthodontic-related videos is still lacking. This study also shows that videos with higher usefulness scores have higher visibility, while in previous studies, there was no difference in visibility between videos with high or low usefulness scores. 25 26 In the future, video uploaders can consider the completeness of the content to make the videos useful, thus increase its visibility. To improve the Indonesian-language orthodontic-related YouTube videos, health workers and educational institutions play a fundamental role in improving the quality, the content comprehensiveness, and the reliability of this information to increase the usefulness and visibility. Collaborations can be made between individual uploaders or influencers and health workers to reach a vast target audience. 18

Although duration has significant effect on viewing numbers, 34 but from the results of this study, most of the videos are more than 6 minutes in length and have higher popularity, quality, and usefulness. This finding is in line with previous research showing that videos with good scores have a longer duration. 10 Additionally, previous research has shown that the videos with the highest usefulness scores are around 7 minutes long. This indicates that the video duration must be long enough to develop the video content, but not too long which can cause viewers to lose attention. 26 This shows that viewers do not care about longer duration as long as the video has good quality and usefulness. If the video has good quality and usefulness, viewers may be more interested in watching the video until the end.

From the aspect of video reliability, it was found that there was no difference in visibility and popularity between videos with high or low reliability. This contradicts previous research showing that high-reliability videos have higher visibility and popularity. 25 These findings indicate that the Indonesian-language orthodontic-related video is still watched despite the low reliability. However, popularity is higher for videos that have high reliability. This shows that the viewer can determine a reliable video as a source of information about orthodontics. Video uploaders can pay attention to clear objectives, remain neutral, include references, and explain if there are still doubts. This aims to improve the video's reliability so that videos can be used as a reliable source of information.

The video with the highest number of views and likes in this study is a video that discusses the orthodontic treatment process. The video is of excellent quality and a good usefulness score. The quality, flow, and completeness of the video information are excellent and very useful to the audience. Besides, the information on the video covers important topics. This is similar to previous findings, stating that the video with the most views and likes is a video with excellent quality. 10 26 However, the video has the highest number of dislikes. This contradicts findings from other studies which showed that videos with the most dislikes had poor usefulness scores. 10 In this study, the videos with the most dislikes also had a higher number of likes and popularity. This shows that many people still liked the video because of its good quality, usefulness, and reliability.

This study has several limitations. YouTube is a dynamic platform that allows videos to be uploaded and deleted at any time, resulting in different search results at different times. 26 To reduce the possibility of bias in this study, video data collection was performed at the same time. In other studies, there is a category of videos uploaded by health companies. 10 However, in this study, there were only nine videos uploaded by health companies. Therefore, videos uploaded by health companies were not included. Further research is needed on videos uploaded by other parties such as health companies and the government.

## Conclusion

Most of the Indonesian-language orthodontics-related videos posted on YouTube were of moderate quality, moderate usefulness, and moderate reliability. Although most of the videos in this study uploaded by individual, significant popularity and reliability were marked among health professionals. Moreover, popularity and usefulness were significant for video more than 6 minutes. Health professional should contribute more quality, usefulness, and reliable health-related videos in social media platform for public seeking information in oral health.

**Appendix A:** Characteristics of top 10 videos based on quality, usefulness, and reliability scores

Video title	Uploader	Number of days since uploaded	Duration (s)	Quality	Usefulness	Reliability	Total
Pakai retainer setelah lepas behel/kawat gigi (Use a retainer after removing the braces)	Professional	319	728	5	7	4	16
Cara kerja kawat gigi (dan kenapa prosesnya lama) (How braces work [and why the process takes so long])	Individual	317	608	5	6	4	15
Kenali macam alat ortodonti (Get to know the types of orthodontic appliances)	Professional	38	184	5	6	4	15
**Part 1 Prosedur** pemasangan kawat gigi/behel—konsultasi, foto gigi, dan cetak gigi (Part 1 Procedure for installing braces/braces—consultation, dental photos, and dental prints)	Individual	196	891	5	6	3	14
Penting! 10 hal yang wajib kamu tahu sebelum pasang behel gigi (Important! 10 things you must know before installing braces)	Professional	57	1,074	5	6	3	14
Mau pakai behel gigi, simak dulu tips dari dokter gigi spesialis ortodonti #dokterbicara (If you want to use braces, take a look at the tips from an orthodontic specialist #doctortalk)	Professional	39	1,082	5	6	3	14
Dokter Gigi Bercerita—Pembahasan seputar ortodonti (kawat gigi) ([Dentist tells story]—discussion about orthodontics [braces])	Professional	258	712	5	6	3	14
Tonton ini sebelum pasang behel! Pengalaman memakai behel (Watch this before putting on braces! Experience wearing braces)	Professional	16	642	4	7	3	14
Pengalaman pasang behel! Harga, proses sampai hasil (Braces experience! price, process, to results)	Individual	168	495	4	6	3	13
Harus berapa lama pakai behel transparan sampai gigi rapi (How long do you have to use transparent braces until your teeth are neat)	Individual	266	430	4	6	3	13

**Appendix B:** Visibility and popularity of top 10 videos

Video title	Uploader	Number of days since uploaded	Durations (s)	Visibility	Popularity	Views	Likes	Dislikes
Pakai retainer setelah lepas behel/kawat gigi (Use a retainer after removing the braces)	Professional	319	728	5,932	2.3	18,925	448	8
Cara kerja kawat gigi (dan kenapa prosesnya lama) (How braces work [and why the process takes so long])	Individual	317	608	267,317	2.5	847,395	21,891	325
Kenali macam alat ortodonti (Get to know the types of orthodontic appliances)	Professional	38	184	186	8.4	71	6	0
Part 1 Prosedur pemasangan kawat gigi/behel—konsultasi, foto gigi, dan cetak gigi (Part 1 procedure for installing braces/braces—consultation, dental photos, and dental prints)	Individual	196	891	69,658	0.72	136,531	1,057	68
Penting! 10 hal yang wajib kamu tahu sebelum pasang behel gigi (Important! 10 things you must know before installing braces)	Professional	57	1,074	2,747	4.09	1,566	64	0
Mau pakai behel gigi, simak dulu tips dari dokter gigi spesialis ortodonti #dokterbicara (If you want to use braces, take a look at the tips from an orthodontic specialist #doctortalk)	Professional	39	1,082	2,500	6.05	975	60	1
Dokter Gigi Bercerita—Pembahasan seputar ortodonti (kawat gigi) (Dentist tells story)—discussion about orthodontics [braces])	Professional	258	712	271	3.00	700	21	0
Tonton ini sebelum pasang behel! Pengalaman memakai behel (Watch this before putting on braces! Experience wearing braces)	Professional	16	642	2,162	15.9	346	55	0
Pengalaman pasang behel! Harga, proses sampai hasil (Braces experience! Price, process, to results)	Individual	168	495	87,216	2.10	146,524	3,157	75
Harus berapa lama pakai behel transparan sampai gigi rapi (How long do you have to use transparent braces until your teeth are neat)	Individual	266	430	3,223	0.7	8,575	63	3
